# Harmful practices in the management of childhood diarrhea in low- and middle-income countries: a systematic review

**DOI:** 10.1186/s12889-015-2127-1

**Published:** 2015-08-18

**Authors:** Emily Carter, Jennifer Bryce, Jamie Perin, Holly Newby

**Affiliations:** 1 Institute for International Programs, Johns Hopkins Bloomberg School of Public Health, 615 North Wolfe Street, Baltimore, MD 21205 USA; 2 grid.420318.c000000040402478XDivision of Policy and Strategy, Data and Analytics Section UNICEF, UNICEF, 3 UN Plaza, New York, NY 10017 USA

## Abstract

**Background:**

Harmful practices in the management of childhood diarrhea are associated with negative health outcomes, and conflict with WHO treatment guidelines. These practices include restriction of fluids, breast milk and/or food intake during diarrhea episodes, and incorrect use of modern medicines. We conducted a systematic review of English-language literature published since 1990 to assess the documented prevalence of these four harmful practices, and beliefs, motivations, and contextual factors associated with harmful practices in low- and middle-income countries.

**Methods:**

We electronically searched PubMed, Embase, Ovid Global Health, and the WHO Global Health Library. Publications reporting the prevalence or substantive findings on beliefs, motivations, or context related to at least one of the four harmful practices were included, regardless of study design or representativeness of the sample population.

**Results:**

Of the 114 articles included in the review, 79 reported the prevalence of at least one harmful practice and 35 studies reported on beliefs, motivations, or context for harmful practices. Most studies relied on sub-national population samples and many were limited to small sample sizes. Study design, study population, and definition of harmful practices varied across studies. Reported prevalence of harmful practices varied greatly across study populations, and we were unable to identify clearly defined patterns across regions, countries, or time periods. Caregivers reported that diarrhea management practices were based on the advice of others (health workers, relatives, community members), as well as their own observations or understanding of the efficacy of certain treatments for diarrhea. Others reported following traditionally held beliefs on the causes and cures for specific diarrheal diseases.

**Conclusions:**

Available evidence suggests that harmful practices in diarrhea treatment are common in some countries with a high burden of diarrhea-related mortality. These practices can reduce correct management of diarrheal disease in children and result in treatment failure, sustained nutritional deficits, and increased diarrhea mortality. The lack of consistency in sampling, measurement, and reporting identified in this literature review highlights the need to document harmful practices using standard methods of measurement and reporting for the continued reduction of diarrhea mortality.

**Electronic supplementary material:**

The online version of this article (doi:10.1186/s12889-015-2127-1) contains supplementary material, which is available to authorized users.

## Background

Diarrheal disease is a leading cause of mortality in children under five, resulting in around 750,000 deaths each year [1]. The WHO recommends first line management of diarrhea in children under five with continued feeding, increased fluids, and supplemental zinc for 10–14 days to prevent dehydration. In addition, the WHO guidelines state that children exhibiting non-severe dehydration should “receive oral rehydration therapy (ORT) with ORS solution in a health facility”. Antimicrobials are recommended only for the treatment of bloody diarrhea or suspected cholera with severe dehydration [2]. The full guidelines, which have evolved over time, are available at http://www.who.int/entity/maternal_child_adolescent/documents/9241593180/en/index.html.

For decades, health initiatives have targeted the expansion of ORS and ORT, including the UNICEF Growth Monitoring, Oral Rehydration, Breastfeeding and Immunization (GOBI) initiative, the USAID/CDC Africa Child Survival Initiative - Combatting Childhood Communicable Diseases (ACSI-CCCD), and the WHO Integrated Management of Childhood Illness (IMCI) initiative. Despite these efforts, a shift in global attention away from diarrhea management seems likely to have contributed to slowing – and even reversals – in progress toward full coverage for ORT [3, 4].

Many fewer programs have specifically targeted non-adherence to other recommended diarrhea management practices, such as the restriction of fluids, breast milk and/or food intake during diarrhea episodes, and incorrect use of modern medicines. All four of these practices are associated with negative outcomes and conflict with WHO treatment guidelines. Curtailment of fluids and restriction of feeding during diarrhea can increase the risk of dehydration, reduce nutritional intake, and potentially inhibit child growth and development. The use of antibiotics and other medications is appropriate only in the treatment of cholera or dysenteric diarrhea in children. Antidiarrheal drugs and some antiemetics not only have no benefit in diarrhea treatment, but may also cause serious, even life-threatening side effects in children [2]. We have referred to these as “harmful practices” from this point forward, understanding that under some circumstances these practices may not be detrimental.

This review summarizes existing literature on harmful practices in diarrhea case management in children under five years of age, including fluid and breastfeeding curtailment, food restriction, and inappropriate use of medications for diarrhea management in children in low- and middle-income countries. The primary objectives of the review are to:

- Determine the documented prevalence of these four harmful practices across low- and middle-income populations, as reported in various studies since 1990;
- Describe how these practices have been examined and reported on previously;
- Explore beliefs, motivations, and contextual factors associated with harmful practices as reported through both quantitative and qualitative studies; and
- Highlight associations between these harmful practices and other characteristics of the episode, child, caregiver, and household.

Findings from this review will identify critical next steps to address harmful practices in diarrhea management and ultimately improve child survival.

## Methods

We searched PubMed, Embase, Ovid Global Health, and the WHO Global Health Library in September 2013. Papers were identified that included variations on the combination of the following terms within the publication’s title or abstract or as a keyword: 1) diarrhea; 2) low- and middle-income country; and one or more terms related to 3) a harmful practice or general management of diarrhea. Search terms were developed in PubMed (see Additional file 1) and translated for the three other databases. Publications were restricted to English-language articles published after 1990.

Quantitative articles were included if the paper reported the prevalence of at least one of the four harmful practices associated with caregiver management of diarrhea in children under the age of five, regardless of study design or representativeness of the sample population. Qualitative articles, or quantitative articles not meeting the quantitative inclusion criteria, were included if they presented substantive findings on beliefs, motivations, or context related to at least one of the four practices in caregiver management of childhood diarrhea. Publications were excluded if they exclusively reported data collected prior to 1990, exclusively reported provider practices, reported findings post-intervention only, or did not specifically focus on treatment of children under 5 years of age. Due to the variety of study designs included in the review, study quality was not formally assessed, because multiple quality assessment frameworks would have been required.

Data extraction was completed by the first author (EC). For all studies, information on the study design, study population, and sample size was extracted. For studies reporting prevalence of practices, data were extracted on the definition of the practice measure, the reported prevalence of the practice, and variation in the practice by other factors (reported as stratified prevalence or odds ratio). For non-prevalence studies, data were extracted related to beliefs, motivations, or context directly related to one or more of the harmful practices and then classified by common themes.

We summarize the results for each of the four harmful practices in the results section of the manuscript. For each practice, we: (1) describe how the practice was defined and measured in these studies; (2) summarize reported findings on prevalence, including variations by characteristics of the diarrhea episode, child, caregiver, and household; and (3) report on beliefs, motivations, and contextual factors investigated and relevant results.

## Results

**Fig. 1:**
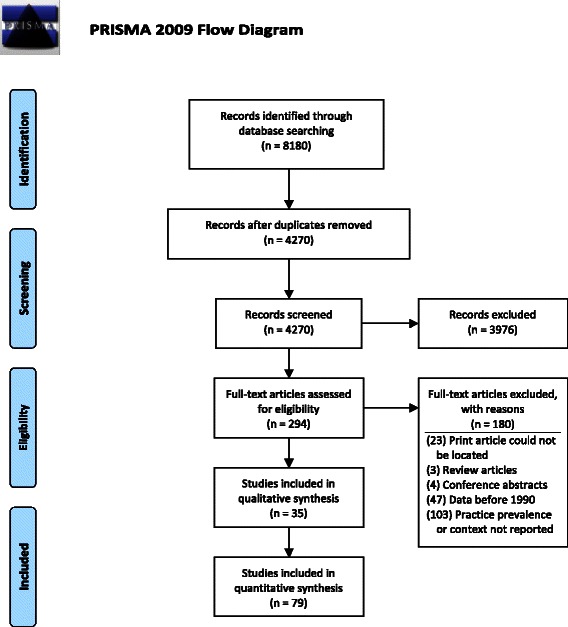
Flow of studies considered in the systematic review

The initial search yielded 2,266 articles in Pubmed, 2,512 articles in Embase, 1,512 articles in Ovid Global Health, and 1,890 articles in the WHO Global Health Library. After removing duplicates, 4,270 unique articles remained. Title and abstract review and full article review were conducted by the first author (EC). After reviewing titles and abstracts, 294 articles were identified for full article review. Based on a review of the full article, 157 articles did not meet the inclusion criteria and a full text copy of 23 manuscripts could not be located. In total, 114 publications met the inclusion criteria and were included in the review (Fig. 1). Of the 79 studies reporting the prevalence of at least one harmful practice, 54 studies utilized a population-based cross-sectional sample (3 nationally representative), 12 studies used a non-cross-sectional design but included a representative population sample, and 13 studies employed a non-representative sample. Of the 35 studies reporting on beliefs, motivations, or context for harmful practices, 9 studies used exclusively qualitative methods, 8 studies used mixed-methods, and 18 studies used exclusively quantitative methods (12 with a representative sample, 6 with a non-representative sample). Although there have been summaries of relevant Demographic and Health Survey (DHS) and Multiple Indicator Cluster Survey (MICS) findings [5, 6], we were unable to identify any country-specific secondary analyses on this topic.

### Study characteristics

**Fig. 2:**
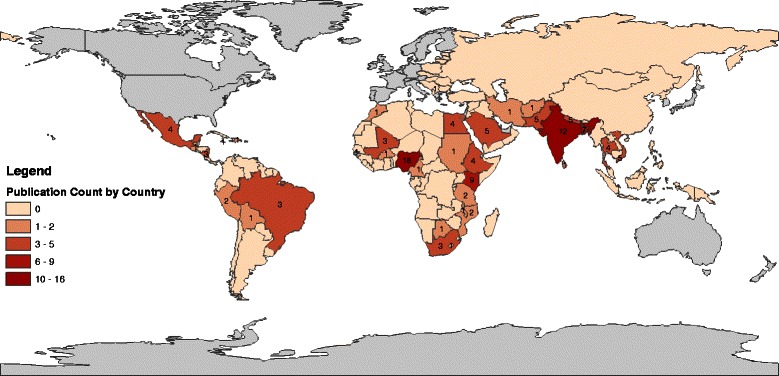
Map with number of studies by country

The publication dates of the 114 studies included in the review were relatively evenly distributed over the period from 1990 to 2013, with publications clustering slightly in the early 1990s and late 2000s/early 2010s. The majority of studies were conducted in South Asia and sub-Saharan Africa (Fig. 2). The number of publications reporting on the prevalence of each of the four practices varied, with the highest proportion reporting on inappropriate medication use (70 %), followed in order of frequency by food restriction (56 %), curtailment of fluids other than breast milk (53 %), and breastfeeding restriction (37 %).

Respondents in the majority of prevalence studies were caregivers of children under 5 years of age, although some studies interviewed mothers exclusively. The age of children referenced for the practice also varied, with the majority of studies referencing children under 5 years of age. The definition of the diarrhea reference episode also varied, ranging from diarrhea in the past 24 h to the most recent diarrhea event, although the most common reference period was the previous two weeks.

### Fluid curtailment

The measurement of fluid intake, and prevalence estimates, varied widely across studies (Table 1, Column 4). Many studies differed in their definition or failed to specify if fluid restriction included or excluded breastfeeding or assessed amount of fluid offered versus consumed. The reported practice of curtailing fluids during a recent episode of diarrhea ranged from as low as 11 % of caregivers in Mirzapur, Bangladesh [7] to over 80 % of caregivers in Kenya’s Nyanza province [8]. Where specified by the study authors, the practice of stopping all fluids was uncommon, generally reported in fewer than 10 % of episodes.

**Table 1:** Prevalence of harmful practices by region and country

Author, Year [reference]	Country	Study design, study population, number of participants	Proportion restricting fluid		Proportion restricting breastfeeding		Proportion restricting food		Proportion using drugs	
Americas										
Emond et al., 2002 [84]	Brazil	Cross-sectional baseline survey preceding intervention, Northeast Brazil 1997, Caregivers of children with diarrhea in the previous 2 days, *n* = 922							Generally give medicines other than ORS	7
Strina et al., 2005 [63]	Brazil	Longitudinal survey, Salvador 1997–1999, Caregivers of children ≤36 months with diarrhea in previous 2 weeks, *n* = 2403 episodes							Gave industrial medicines	40.9
									Gave industrial medicines & home preparation	2.7
Webb et al., 2010 [85]	Guatemala	Longitudinal survey, Population of Spanish-Mayan Descent 1996–1999, Caregivers of children <36 months with diarrhea in previous 19 days, *n* = 466	Stopped or less fluid^a^	55	Stopped or less breastfeeding^b^	26.6	Stopped or less food	15		
Bachrach et al., 2002 [21]	Jamaica	Case-control hospital based survey, Kingston 2007, Caregivers of children <5 years presenting at hospital, *n* = 215 total, 117 gastroenteritis cases							Child presenting with gastroenteritis: Gave antidiarrheal/ antimotility drug before coming to hospital	36
Martinez et al., 1991 [52]	Mexico	Cross-sectional survey, Rural Highlands of Central Mexico (year not specified), Caregivers of children <5 years, diarrhea episode reference unclear, *n* = 38							Give pill as first treatment for diarrhea	47
									Give over-the-counter drug to child	53
Perez-Cuevas et al., 1996 [40]	Mexico	Cross-sectional survey, Tiaxcala (year not specified), Caregivers of children <5 years with diarrhea in previous 2 weeks, *n* = 747	“Withheld” non-breast milk	27.2	Stopped breastfeeding^b^	12.2	Stopped or reduced food other than milk	9.1	Treated with any drug	35.2
			No liquids given	3			Any dietary restriction	36.6		
Martinez et al., 1998 [86]	Mexico	Cross-section of ethnographic study participants, 3 States (year not specified), Caregivers of children <5 years in reference to most recent diarrhea episode, *n* = 186							Gave antimicrobial	37.1
									Gave antidiarrheal	28
									Gave antipyretic	18
Smith et al., 1993 [51]	Nicaragua	Cross-sectional survey, Rural Pacific Coastal Plain (year not specified), Caregivers of infants, diarrhea episode reference unclear, *n* = 70			Stopped breastfeeding (among those who reported changing feeding)^b^	4	Did not give solid foods (among those who reported changing feeding)^c^	13		
Gorter et al., 1995 [79]	Nicaragua	Cross-section of ethnographic study participants, Rural Pacific Coastal Plain 1990, Caregivers of children <5 years with diarrhea in previous 2 weeks, *n* = 216							Gave antibiotic	22
									Gave parasite medicine	19
									Gave laxative	6
Vazquez et al., 2002 [33]	Nicaragua	Cross-sectional survey, North of Central Region 1990, Caregivers of children <5 years with diarrhea in previous 2 weeks, *n* = 187					Child ate less than usual	43.5	Gave any pharmaceutical	60
Kristiansson et al., 2009 [87]	Peru	Cross-sectional survey, Yurimaguas and Moyobamba Departments 2002, Caregivers of children 6–72 months with illness in previous 2 weeks, *n* = 780							Antibiotic use reported by wealth quintile only	
Europe										
Berisha et al., 2009 [16]	Kosovo	Cross-sectional survey, Kosovo 2005, Mothers of children <5 years in reference to most recent diarrhea episode, n = 107	Less fluid or none^a^	62.6	Stopped or reduced amount of food or breastfeeding	43.9				
			Same fluids^a^	19.6	Same amount of food or breastfeeding	48.6				
Eastern Mediterranean										
Azim et al., 1993 [37]	Afghanistan	Cross-sectional study, Paktika Province 1991, Caregivers of children <5 years with diarrhea in previous 2 weeks, *n* = 87	Same or less fluid^d^	43.7	Stopped breastfeeding^b^	5.9	Stopped or less food	33.5	Gave any drug	66
Langsten et al., 1994 [88]	Egypt	Longitudinal survey, Lower Egypt 1990, Caregivers of children <5 years with diarrhea in previous 2 weeks, *n* = 4900	Stopped fluids other than BF and milk^d^	2.8	Stopped breastfeeding^b^	2.5	Stopped food	5.8		
			Reduced other fluids^d^	10.9	Decreased breastfeeding^b^	11.9	Reduced food	22.7		
			Reduced non-breast milk^d^	15.3						
			Stopped non-breast milk^d^	9.9						
Langsten et al., 1995 [57]	Egypt	Longitudinal survey, Lower Egypt 1990–1991, Caregivers of children <5 years with diarrhea in previous 2 weeks, n = 4900							Among acute non-dysenteric cases: Used antibiotics	46.5
									Among acute non-dysenteric cases: Used antibiotics only	3.2
									Among acute non-dysenteric cases: Used other medicine	63.3
									Among acute non-dysenteric cases: Used other medicine only	18.6
									Among all cases: Used antibiotics	45.6
									Among all cases: Used antibiotics only	3.4
									Among all cases: Used other medicine	63.0
									Among all cases: Used other medicine only	19.3
Jousilahti et al., 1992 [75]	Egypt	Cross-sectional cluster study, Lower Egypt 1992, Caregivers of children <5 years with diarrhea in previous 24 h, n = 766	Same or less fluid^d^	75.6	Stopped breastfeeding^b^	3.7	Stopped or less solid or semi-solid food	30.2	Gave any drug	54.2
									Gave drug and ORS	17.6
									Gave drug but no ORS	36.5
El-GIlany et al., 2005 [62]	Egypt	Cross-sectional study, Dakahalia 2002–2003, Caregivers of children <5 years with diarrhea in previous 2 weeks, *n* = 1052	Same or less fluid^e^	29			Stopped feeding^e^	12.7	Gave any drug	74.7
									Among those receiving a drug:	36.9
									Antibiotic^f^	73.9
									Antidiarrheal^f^	73.9
									Antiemetic^f^	16.7
									Antiprotozoal^f^	5.7
									Antipyretic^f^	9.6
									Antispasmodic^f^	1.7
Amini-Ranjbar et al., 2007 [53]	Iran	Cross-sectional study, Kerman 2005, Caregivers of children 6–24 months with diarrhea in previous 2 months, *n* = 330			Same or less breastfeeding^g^	53.8	Decreased solid foods	20		
WHO, 1991 [89]	Morocco	Cross-sectional study, National 1990, Caregivers of children <5 years with diarrhea in previous 24 h, *n* = 1066	Same or less fluid^e^	70					Gave any drug	22.6
Morisky et al., 2002 [90]	Pakistan	Cross-sectional survey, National 1991–1992, Caregivers of children <2 years in reference to most recent episode, *n* = 5433	Stop fluids^e^	9.2			Stopped food	5.9	Gave antibiotic	11
							Reduced food	6.2	Gave other medicine	9.2
Quadri et al., 2013 [13]	Pakistan	Cross-sectional study (HUAS), Low-Income peri-urban area near Karachi 2007, Caregivers of children <5 years with diarrhea in previous 2 weeks, *n* = 959	Did not offer “to drink” (at home before seeking care)^e^	22.5			Did not offer “to eat” (at home before seeking care)^c^	44.1	Gave antibiotic (at home)	7.7
Nasrin et al., 2013 [91]	Pakistan	Cross-sectional study (HUAS), Low-Income periurban area near Karachi 2007, Caregivers of children <5 years with diarrhea in previous 2 weeks, *n* = 349	Offered same or less than usual to drink	33.9			Offered less than usual to eat^e^	33.6		
Bella et al., 1994 [92]	Saudi Arabia	Case–control study, Eastern Province (year not specified), Caregiver of infant with diarrhea at time of survey versus caregiver of infant without diarrhea, *n* = 344 total, 68 cases	Stopped bottle feeding (among cases who were bottle feeding)	35						
al-Mazrou et al., 1995 [93]	Saudi Arabia	Cross-sectional survey, National 1991, Caregivers of children <5 years with diarrhea in the previous 2 weeks, *n* = 6300 screened							Gave drugs	40.7
									Gave IV fluids	4.7
Bani et al., 2002 [12]	Saudi Arabia	Cross-sectional hospital based survey, Riyadh City (year not specified), Mothers of children ≤24 months with diarrhea attending primary health clinic, *n* = 237	Less fluid given^e^	11.3	Less frequency of breastfeeding^b^	24.6	Less solid/semi-solid food given	22.7		
			Same fluid given^e^	13.2	Same frequency of breastfeeding^b^	37.7	Same solid/semi-solid food given	22.6		
Moawed et al., 2000 [20]	Saudi Arabia	Cross-sectional hospital based survey, Riyadh City 1998, Mothers of infants with diarrhea attending 2 pediatric hospital diarrhea centers, *n* = 300			Stop breastfeeding or milk feeding	62			Gave unprescribed medicine	38
Africa										
Wilson et al., 2012 [11]	Burkina Faso	Cross-sectional survey, Orodara Health District 2012, Primary caregivers of children <27 months with diarrhea in previous 2 weeks, n = 1067	Same or less fluid^e^	64.1	Stopped breastfeeding^b^	1.2	Stopped or decreased feeding normal diet^e^	53.2	Gave any drug other than ORS	41.2
									Gave antibiotic or unidentified drug	27.6
Olango et al., 1990 [17]	Ethiopia	Cross-sectional survey, Rural population in Wolayta district (year not specified), Mothers of children <5 years with diarrhea in previous 2 weeks, *n* = 619	Stopped fluids (breastfed children separate category within fluid intake measure)	8.6			Stopped food (not weaned are additional category)	15.2	Gave injection	40.8
			Decreased fluids	42.3			Decreased food	54.4	Gave tablets	19.6
			Same amount of fluids	10.3			Same amount of food	10.2		
Ketsela et al., 1991 [94]	Ethiopia	Cross-sectional survey, Shewa Administrative Regions 1990, Mothers of children <5 years, diarrhea episode reference unclear, *n* = 750	No fluids^a^	26.8	No breastfeeding^g^	3.5	Gave less fluid than^c^	35.9		
			Less than usual fluid^a^	31.4			Gave same fluid as usual^c^	38.2		
			Same as usual fluid^a^	23.8			Gave no food^c^	10.5		
Mash et al., 2003 [95]	Ethiopia	Cross-sectional survey, Oromia Region 1997, Caregivers of children <24 months with diarrhea in the previous fortnight, *n* = 111	Stopped or decreased fluids^a^	47.7	Stopped or decreased breastfeeding^b^	67.6	Stopped or less solid or semi-solid food	67.6		
Mediratta et al., 2010 [9]	Ethiopia	Case–control hospital based study, Gondar 2007, Caregivers of children <5 years with diarrhea attending referral hospital, case n = 220	Less of other fluids^a^	29	Gave less breast milk^b^	24	“Withheld” food	46		
			Same amount^a^	44	Same amount of breast milk^b^	34				
Saha et al., 2013 [96]	Gambia	Cross-sectional survey, Upper River Region 2009, Caregivers of children <5 years with diarrhea in the previous 2 weeks, *n* = 258	Same or less fluid^e^	36.1			Less than usual amount of food	72.5	Gave antimicrobial (at home)	9.7
									Gave antimicrobial (among those seeking care at health facility)	18.6
									Gave injectable medicine (among those seeking care at health facility)	43.7
Oyoo et al., 1991 [39]	Kenya	Cross-sectional survey, 6 sites across Kenya 1990, Caregivers of children <5 years with diarrhea in the previous 2 weeks, *n* = 23884 screened	Same or less fluid^e^	74 - 96	Stopped breastfeeding^b^	0-3.1	Stopped feeding^a^	19.5 - 53.3	Gave any drug (range across clusters)	25.9 - 47.1
Mirza et al., 1997 [97]	Kenya	Longitudinal study with 24 h dietary recall, Kibera Slum 1989–1990, Caregivers of children 3–37 months with diarrhea in the previous 3 days, *n* = 1496 episodes	Gave less cow’s milk than before diarrhea	28.7						
Othero et al., 2008 [7]	Kenya	Longitudinal study, Nyanza Province 2004–2006, Caregivers of children <5 years in reference to most recent episode, *n* = 927	Offered nothing to drink^e^	20.5			Did not eat anything (among all children)	39	Gave anti-diarrheal drugs	45.3
			Offered much less^e^	59.9						
			Offered somewhat less^e^	3.3						
			Offered same^e^	5.3						
Burton et al., 2011 [98]	Kenya	Cross-sectional survey, Rural Western Kenya 2005, Caregivers of children <5 years with diarrhea in the previous 2 weeks, *n* = 188							Gave antibiotic	62.4
									Gave antimalarial	52.4
									Gave IV fluid	2.6
Olson et al., 2011 [42]	Kenya	Cross-sectional survey, Asembo (*n* = 371) and Kibera (*n* = 389) 2007, Caregivers of children <5 years with diarrhea in the previous 2 weeks	Asembo: Stopped fluids other than breast milk and porridge (among those giving fluids in week before illness)	9	Asembo: Stopped breastfeeding^b^	5	Asembo: Stopped porridge	9	Asembo: Gave oral medication (not ORS or herbs)	77
			Kibera: Stopped fluids other than breast milk and porridge	18	Kibera: Stopped breastfeeding^b^	16	Kibera: Stopped porridge	36	Kibera: Gave oral medication (not ORS or herbs)	81
			Asembo: Decreased fluids^h^	42	Asembo: Decreased breastfeeding^h^	32	Asembo: Decreased porridge^h^	54	Asembo: Gave injected medication	24
			Kibera: Decreased fluids^h^	47	Kibera: Decreased breastfeeding^h^	47	Kibera: Decreased porridge^h^	69	Kibera: Gave injected medication	28
			Asembo: Same fluids^h^	47	Asembo: Same breastfeeding^h^	59	Asembo: Same porridge^h^	41	Asembo: Gave IV fluids	8
			Kibera: Same fluids^h^	22	Kibera: Same breastfeeding^h^	28	Kibera: Same porridge^h^	18	Kibera: Gave IV fluids	7
							Asembo: Stopped soft or solid food	10		
							Kibera: Stopped soft or solid food	37		
							Asembo: Decreased solid food^h^	54		
							Kibera: Decreased solid food<	70		
							Asembo: Same solid food^h^	41		
							Kibera: Same solid food^h^	23		
							Asembo: Stopped or Decreased feeding (including BF, porridge, solids)	36		
							Kibera: Stopped or Decreased feeding (including BF, porridge, solids)	54		
Omore et al., 2013 [41]	Kenya	Cross-sectional survey (HUAS), Western Kenya 2007, Caregivers of children <5 years with diarrhea in the previous 2 weeks, *n* = 275	Offered same amount to drink	19			Offered usual amount to eat	16		
			Offered less to drink	67			Offered less to eat	83		
			Among those offering less: Somewhat less	52			Among offering less: Somewhat less	33		
			Much less	38			Much less	30		
			Nothing	10			Nothing	37		
Nasrin et al., 2013 [91]	Kenya	Cross-sectional survey (HUAS), Western Kenya 2007, Caregivers of children <5 years with diarrhea in the previous 2 weeks, *n* = 275							Gave leftover antibiotics at home	16
Zwisler et al., 2013 [68]	Kenya	Cross-sectional survey, 4 Provinces 2012, Caregivers of children <5 years with diarrhea in the previous 2 months, *n* = 857							Gave antibiotic	51.3
									Gave antimotility agent	10.4
Simpson et al., 2013 [99]	Kenya	Cross-sectional survey, Western Kenya (year not specified), Caregivers of children 6–60 month with diarrhea in the previous 6 months, *n* = 100							Gave antibiotic (at any point)	64
									Gave antimotility (at any point)	13
									Gave antibiotic (1^st^ treatment)	26
									Gave antibiotic (1^st^ or 2^nd^ treatment)	46
Winch et al., 2008 [71]	Mali	Cross-sectional baseline survey preceding intervention, Southern Mali 2004, Caregivers of children <5 years with diarrhea in the previous 2 weeks, n = 228	Same or less fluid or breast milk	82.7					Gave antibiotics	57
			Stopped feeding or breastfeeding	46					Gave metronidazole	7.5
									Gave antidiarrheal	2.6
									Among children with only diarrhea symptoms gave: Antibiotic	16
									Antimalarial	16
									Paracetamol	10
Perez et al., 2009 [100]	Mali	Cross-sectional survey in intervention comparison area, Mopti Region 2006, Caregivers of children <5 years, reference episode unclear, *n* = 401							Gave any drug	56.1
Nasrin et al., 2013 [91]	Mozambique	Cross-sectional survey, Rural Southern Mali 2007, Caregivers of children <5 years with diarrhea in the previous 2 weeks, *n* = 67					Offered less than usual to eat	38.3	Gave leftover antibiotics at home	3.6
Nhampossa et al., 2013 [15]	Mozambique	Cross-sectional study (HUAS), Rural Southern Mozambique 2007 (Study 1 *n* = 67) and 2009–2012 (Study 2 *n* = 246), Caregivers of children <5 years with diarrhea in previous 2 weeks	Study 1: Reduced or stopped breastfeeding/usual fluid intake	12					Study 1: Gave antibiotic (Among those seeking treatment)	14
			Study 1: Maintained same fluid or breast milk intake	73						
			Study 2: Reduced or stopped breastfeeding/usual fluid intake	79						
			Study 2: Maintained same fluid or breast milk intake	1						
Ekanem et al., 1990 [47]	Nigeria	Diarrhea surveillance survey, Periurban Lagos (year not specified), Mothers of children 6–36 months, reference episode is general case, *n* = 200			Normal breastfeeding pattern continued^b^	76.9				
					Decreased breastfeeding^b^	10.4				
Babaniyi et al., 1994 [10]	Nigeria	Cross-sectional study, Suleja 1991, Caregivers of children <5 years with diarrhea in previous 2 weeks, *n* = 340	Normal amount of “other” fluids^ai^	55.6	Stopped breastfeeding^b^	7.7	Stopped or less solid food	42.4	Gave any drug (at home)	53.5
			Less “other” fluids^ai^	22.6						
Okoro et al., 1995 [74]	Nigeria	Cross-sectional study, Cross River State 1994, Caregivers of children <5 years with diarrhea in previous 24 h, *n* = 488							Gave any drug	75.6
									Gave drug and ORS/SSS	51.9
Okunribido et al., 1997 [26]	Nigeria	Longitudinal study, Rural Yoruba communities of rural Oyo State (year not specified), Caregivers of children <5 years with diarrhea in previous 2 weeks, *n* = 98	Stopped fluids (among those who noticed fluid intake)^e^	2	Child could not suck	23.4	Stopped food	3	Gave Western medicine: 1^st^treatment, among those treating	37.7
			Child refused fluid	29.5	Lost appetite	34.6	Reduced appetite	68.8	Gave Western medicine: 2^nd^treatment, among those treating	30.3
									Gave Western medicine at any point for watery diarrhea	50
									Gave Western medicine at any point for presumed dysentery	52.7
Edet et al., 1996 [101]	Nigeria	Cross-sectional study, Oduknani 1994, Caregivers of children <5 years with diarrhea in previous 24 h, *n* = 5296 screened	Less fluid^a^	48.2	Stopped breastfeeding^b^	59.9	Stopped feeding	13.8		
			Same fluid^a^	37.3			Less food	32.8		
							Same food	49		
Omokhodion et al., 1998 [102]	Nigeria	Cross-sectional study, Market women in Ibadan 1996–1997, Market women with children <5 years in reference to any diarrhea episode, Bodia *n* = 266, Gbagi *n* = 260							Bodija Market: Went to chemist to buy drugs	12
									Gbagi Market: Went to chemist to buy drugs	19
									Bodija Market: Used drugs prescribed for previous illness	7
									Gbagi Market: Used drugs prescribed for previous illness	5
Ene-Obong et al., 2000 [81]	Nigeria	Surveillance study, Market women in Enugu State 1993–1994, Market women with children <5 years with diarrhea in previous 2 weeks, *n* = 80							Gave pharmaceutical	28.8
									Gave pharmaceutical & sugar-salt solution	33.8
Omotade et al., 2000 [38]	Nigeria	Surveillance study, Oyo State 1993–1994, Caregivers of children <5 years with diarrhea in previous week, *n* = 158							Gave antimicrobial	46.8
Uchendu et al., 2009 [60]	Nigeria	Cross-sectional hospital based study, Enugu 2006, Caregivers of children <5 years attending health clinic with diarrheal disease and vomiting, *n* = 156							Gave antibiotic (at home)	51.3
									Gave antimotility/antidiarrheal (at home)	44.9
Uchendu et al., 2011 [45]	Nigeria	Cross-sectional hospital based study, Enugu 2006, Caregivers of children <5 years attending health clinic with diarrheal disease and vomiting, *n* = 156					Stopped feeds^e^	5.2		
Ogunrinde et al., 2012 [103]	Nigeria	Cross-sectional hospital based survey, Northwestern Nigeria (year not specified), Caregivers of child 1–59 months attending health clinic with diarrheal disease, *n* = 186							As first line treatment gave:	
									Antibiotic	23.7
									Antidiarrheal	12.7
									ORS, antibiotic, antidiarrheal	3
Ekwochi et al., 2013 [64]	Nigeria	Cross-sectional hospital based study, Enugu 2012, Caregivers of children ≤5 years attending university teaching hospital, reference any diarrhea episode, *n* = 210							Gave unprescribed antibiotic	46.7
Cooke et al., 2013 [104]	South Africa	Cross-sectional hospital based study, Capetown 2007–2008, Caregivers of children <65 months with severe diarrhea attending hospital, *n* = 142	Same or less fluid among all (but gave some ORS or milk)	36.6	Stopped breastfeeding/milk (but gave other fluids)^b^	35.2				
Haroun et al., 2012 [105]	Sudan	Cross-sectional hospital based study, Gezira (year not specified), Mothers of children <5 years, diarrhea episode reference unclear, *n* = 110	Stopped or reduced fluid during episode^e^	49			Stopped feeding^e^	30		
			Same amount of fluid during episode^e^	33						
			Stopped or reduced fluid during episode but didn’t change amount of food^e^	23						
Kaatano et al., 1997 [8]	Tanzania	Cross-sectional survey, North-western lake districts (year not specified), Caregivers of children <5 years with diarrhea in previous 2 weeks, *n* = 89	Stopped or decreased fluid^e^	12.6	Stopped breastfeeding^b^	46.7	Stopped or decreased food	13.8	Gave anti-diarrheal	29.2
									Gave antibiotic	13.5
South East Asia										
Alam et al., 1998 [82]	Bangladesh	Cross-sectional survey, Metropolitan Chittagong 1996–1997, Caregivers of children <5 years with diarrhea in previous 2 weeks, *n* = 360							“Inappropriate or non-recommended drug use” among those receiving treatment	73.5
									Gave metronidazole (denominator all consultations)	38.6
									Gave antibiotic (denominator all consultations)	17.5
									Gave antiemetic (denominator all consultations)	12.2
									Gave antidiarrheal (denominator all consultations)	8
Ali et al., 2000 [27]	Bangladesh	Cross-sectional survey, Brahmanharia district 1993, Caregivers of children <5 years with diarrhea in previous 24 h, *n* = 186	Drank less than usual amount of water (not amount offered)	17						
Taha et al., 2002 [106]	Bangladesh	Cross-sectional survey, Cox’s Bazar district 1994, Mothers of children <5 years, diarrhea episode reference unclear, *n* = 297	No fluids for treating diarrhea^e^	11.7	Stopped breastfeeding^b^	11.7	Did not give solid or semi-solid food^c^	40.4		
Baqui et al., 2004 [73]	Bangladesh	Community based controlled trial, Matlab 1998–2000, Caregivers of children 3–59 months with diarrhea in previous week, *n* = 297							Gave antibiotic	34.3
									Gave other medicine	44.8
									Gave IV	0.3
Larson et al., 2009 [107]	Bangladesh	Cross-sectional baseline survey preceding intervention, Dhaka 2006, Caregivers of children 6–59 months with diarrhea in previous 2 weeks, *n* = 640							Gave antibiotic	34.7
Das et al., 2013 [14]	Bangladesh	Cross-sectional survey (HUAS), Rural Mirzapur 2007, Caregivers of children <5 years with diarrhea in previous 2 weeks, *n* = 1128	Offered less than usual amount of fluids	10.8			Offered less to eat (at home before seeking care)	28.7	Gave antibiotics (at home before seeking care)	2.4
			Same amount	61.3						
			Same or less	72.1						
Sood et al., 1990 [108]	India	Cross-sectional survey, Rural Haryana State (year not specified), Caregivers of children <5 years, reference any diarrhea episode, *n* = 108			Generally stopped breastfeeding	0	Some food restricted	83.33		
Rasania et al., 1993 [23]	India	Cross-sectional survey, New Delhi (year not specified), Caregivers of children <5 years, diarrhea episode reference unclear, *n* = 254			Restricted breastfeeding^b^	12.59	Gave less food during convalescence	26.38		
					Stopped breastfeeding^b^	19.29	Shifted from solid to liquid diet	45.27		
							Stopped all food^e^	9.84		
							Restricted “few” foods	16.53		
Gupta et al., 2007 [109]	India	Cross-sectional survey, Urban Delhi slum 2004, Caregivers of children <5 years with diarrhea in previous 2 weeks, *n* = unclear 1307	Stopped fluid^e^	20			Stopped feeding (not clear if food or breastfeeding)	50		
Ahmed et al., 2009 [46]	India	Cross-sectional survey, Kashmir Valley 2006, Caregivers of children <5 years with diarrhea in previous 24 h (*n* = 1055) and 2 weeks (*n* = 2836)					Among diarrhea in 15 days: Feeding restricted^e^	4	Diarrhea in last 24 h: Gave antibiotic	77.9
							Diarrhea in last 24 h: Feeding restricted^e^	6.9		
Shah et al., 2012 [31]	India	Cross-sectional survey, Urban slum of Aligarh 2009, Caregivers of children <5 years with diarrhea in previous 2 weeks, *n* = 101			Stopped or decreased breastfeeding (among EBF 0-6 m)^b^	30.77	Interrupted, stopped or decreased feeding (among not breastfeeding: 7 m-5 years)	37.8		
					Stopped or decreased breastfeeding (among non-EBF 0-6 m)^b^	80				
Zwisler et al., 2013 [68]	India	Cross-sectional survey, 7 States 2012, Caregivers of children <5 years with diarrhea in the previous 2 months, *n* = 988							Gave antibiotic	56.4
									Gave antimotility agent	3
WHO 1991 [110]	Nepal	Cross-sectional survey, Terai (*n* = 335) and Midhills (*n* = 526) 1990, Caregivers of children <5 years with diarrhea in previous 24 h	Terai: Same or less fluid^a^	72	Terai: Stopped breastfeeding^b^	1	Terai: Stopped or Less Feeding	25	Terai: Gave drug, no ORS	21.5
			Midhills: Same or less fluid^a^	91	Midhills: Stopped breastfeeding^b^	1	Midhills: Stopped or Less Feeding	39	Midhills: Gave drug, no ORS	14.3
									Terai: Gave drug and ORS	4.5
									Midhills: Gave drug and ORS	4.9
Jha et al., 2006 [111]	Nepal	Cross-sectional hospital based study, Sunsari District (year not specified), Caregivers of children <5 years with diarrhea attending PHC, *n* = 330					Not Given Food^ec^	2.1	Gave any drug at any point	70
							Less frequency of food given^ec^	12.5	Gave antibiotic	19.9
							More liquid mixed food given	13.1	Gave antimotility drug	16.8
							Fed as usual, child refused	14.6	Gave anti-vomiting drug	15.5
							Usual feeding	57.7	Gave IV	17.7
WHO 1993 [77]	Sri Lanka	Cross-sectional survey, North-western Province 1992, Caregivers of children <5 years with diarrhea in previous 2 weeks, *n* = 10077 screened	Same or less fluid^e^	63			Stopped feeding^e^	23	Gave any medicine	71
Wongsaroj et al., 1991 [65]	Thailand	Cross-sectional survey, 12 Regions 1991, Caregivers of children <5 years with diarrhea in previous 2 weeks, *n* = 733	Same or less fluid^e^	91.8	Stopped breastfeeding^b^	16.6	Stopped solid foods	28.7	Gave any antibiotic or antidiarrheal	58.6
									Gave IV	6.2
									Gave antibiotic	18
									Gave antidiarrheal	19.3
									Gave both antibiotic and antidiarrheal	21.3
Prohmmo et al., 2006 [28]	Thailand	Surveillance survey, Northeast Region 2002, Caregivers of children <5 years with diarrhea in previous 2 weeks, *n* = 47 episodes	Same or decreased fluid	42.5	Stopped breastfeeding^b^	0			Gave antimicrobial	45
									Gave antiemetic	19
									Gave antidiarrheal	13
									Gave cold medicine	15
									Gave antipyretic	25
Western Pacific										
Dearden et al., 2002 [22]	Vietnam	Cross-sectional survey, Rural northern province, Caregivers of children 6–18 months, reference any diarrhea episode, *n* = 100					Generally give less or no foods and liquids	71		
Hoan et al., 2009 [112]	Vietnam	Cross-sectional survey, Rural district (year not specified), Caregivers of children <5 years with diarrhea in previous 2 weeks, *n* = 133							Among children with only diarrhea symptoms gave:	54.1
									Antibiotics	36.1
									Anti-diarrheal	36.1
									Antihistamine	3
									Analgesic/antipyretic	13.5
									Cough and cold prep	0.8
									Corticosteroid	2.3

^a^Excluding breast milk

^b^Among those breastfeeding

^c^Unclear if only among those receiving solid or semi-solid food before illness

^d^Among drinking fluids other than breast milk

^e^Inclusion/exclusion of breastfeeding not specified

^f^Among those receiving drug as treatment

^g^Unclear if only among those breastfeeding at time of illness

^h^Among those who continued to receive fluids; breast milk; food

^i^Explicitly excluding ORS/SSS

Multiple studies explored variations in fluid curtailment by characteristics of the diarrhea episode, child, caregiver, and household (Table 2). Fluid curtailment was associated with diarrhea severity and vomiting in two studies [9, 10], whereas increase in fluid was associated with long illness duration and poor appetite [11]. Studies in Pakistan, Bangladesh, and Saudi Arabia found no clear association between fluid restriction and the age of the child [12–14]. However, a study in Mozambique reported that less fluid was given to infants relative to older children [15]. Younger mothers and mothers who did not work outside the home [12] and less educated mothers [16] were more likely to curtail fluids.

**Table 2:** Factors associated with harmful practice

Level	Factor	Positive association (harmful practice more likely)	Negative association (harmful practice less likely)	No association	No test of significance
Association with fluid curtailment					
Episode	Dehydrated (vs not dehydrated)				[57]
	Severe disease	[10]			[57]
	Child vomited (vs did not vomit)	[9]			
	Child was anorexic		[11]		
	Longer duration of episode		[11]		
Child	Older child age		[15]	[12]	[13, 14]
Caregiver	Older maternal age		[12]	[16]	
	Higher maternal education		[16]	[12]	
	Older maternal age at marriage			[12]	
	Caregiver employed		[12]		
Household	Live in urban area (vs rural)			[16]	[95]
Association with breastfeeding restriction					
Episode	Dehydrated (vs not dehydrated)				[57]
	Severe disease				[57]
Child	Older child age			[12]	
Caregiver	Older maternal age		[12]		
	Higher maternal education		[12]		
	Older maternal age at marriage			[12]	
	Caregiver employed			[12]	
Household	Live in urban area (vs rural)			[33]	[95]
Association with Food Restriction					
Episode	Dehydrated (vs not dehydrated)	[40]			[57]
	Severe disease	[40]			[57]
	Child had fever	[11]			
	Child was anorexic	[11]			
	ORS use	[41]			
	Sought care outside home	[41]			
Child	Older child age	[42]		[12]	[13, 14]
Caregiver	Older maternal age			[12, 16]	[90]
	Higher maternal education		[12, 16]		[90]
	Older maternal age at marriage			[12]	
	Caregiver employed			[12]	
Household	Greater household income				[90]
	Live in urban area (vs rural)			[16]	[90, 95]
Association with inappropriate drug use					
Episode	Dehydrated (vs not dehydrated)	[60]		[40]	[57]
	Severe disease			[10, 40]	[57]
	Longer disease duration	[63]			
	Classification of diarrhea				[81]
	ORS use	[60, 63]			[68]
	Sought care outside home	[11, 41]			
Child	Older child age				[13, 14]
Caregiver	Higher maternal education		[64]	[60]	
Household	Greater household income			[60, 87]	
	Live in urban area (vs rural)				[93]

Multiple studies have attributed the practice of fluid curtailment to caregiver beliefs about the impact of fluid intake on a child’s diarrhea episode (Table 3). Multiple studies reported that caregivers often stated that more or specific fluids would increase the severity of the illness [17–19] or could not be digested [20–22]. Two studies suggested these beliefs were informed by caregivers’ observations that reduced fluids decreased stool output and diarrhea intensity [7, 23]. One study reported that certain types of diarrhea are perceived to be manageable by adjusting fluid intake, while others require traditional or spiritual methods, or no treatment at all [24]. The beliefs of family and community members, particularly elderly relatives, have also been reported as influential in determining caregiver practices related to fluids and feeding during childhood diarrhea episodes [22, 24, 25]. In three studies caregivers reported reduced fluid intake due to child refusal, child crying, or decreased thirst [22, 26, 27]. In one study, mothers reported they did not encourage increased fluids because they were inexperienced in how to do this [27].

**Table 3:** Beliefs, motivations, and context related to harmful practices by region and country

Author, Year [reference]	Country	Study design: methods (number conducted), study population	Source of information on diarrhea treatment	Expected effect of treatment	Restriction of specific food or fluid	Treatment specific to type or cause of diarrhea	Drug specific: strength/effectiveness	Drug specific: and source/availability	Other
Americas									
Hudelson et al., 1994 [44]	Bolivia	Qualitative study: Indepth interviews IDIs (65), hypothetical case scenarios (10), and observation (5) of mother and health workers, El Alto 1993, Mothers of children <5 years and health workers		Food: Mothers worry increasing food intake could worsen episode		General: Type of treatment sought is dependent on perceived cause of the illness			Feeding: Diet is already poor so doesn’t vary much during episode
				Food: Some may offer less food to reduce stool output		Drugs: Drugs are used to treat “diarrea por infeccion”			Food: Reduction in intake due to loss of appetite. Caregivers unaccustomed to encouraging feeding.
Larrea-Killinger et al., 2013 [113]	Brazil	Qualitative study: IDIs (29) and observations, Salvador 1997–2004, Mothers and grandmothers of children <5 years					Combination of ORS and antibiotics believed to reduce severity of episode		
McLennan et al., 2002 [49]	Brazil	Qualitative study: IDIs (29) and observations, Salvador 1997–2004, Mothers and grandmothers of children <5 years			Feeding: 1/3 mothers reported restricting some foods				Drugs: 73 % mothers believe child should be given antibiotic for episode
					Feeding: 95 % believe at least one food item should be restricted				
					Food: 38 % believe all solid foods should be restricted				
					BF: Few (3 %) believe BF should be suspended				
Granich et al., 1999 [114]	Dominican Republic	Quantitative study: Structured interviews (582), Periurban Santo Domingo 1996, Mothers of children <5 years							Drugs: 71 % of caregivers would give pill or injection for hypothetical episode of diarrhea
Ecker et al., 2013 [115]	Peru	Quantitative study: Structured interviews (1200), Periurban Lima (year not specified), Caregivers of children <5 years							Drugs: 65 % of caregivers believe antibiotic is necessary to treat hypothetical case of non-dysenteric diarrhea
Europe									
Eastern Mediterranean									
Ali et al., 2003 [50]	Pakistan	Quantitative study: Self-administered questionnaire (400), Karachi 2000, Adult females attending clinic	Food: Most caregivers reported receiving information on food restriction from mother or grandmother		Food: Heavy foods, bread, meat commonly restricted				
					Food: 2 % of women believe all food items should be restricted				
Agha et al., 2007 [116]	Pakistan	Quantitative study: Structured interview (647), Gambat, Singh Province (year not specified), Caregivers of children 6–59 months			Fluid: 12 % of caregivers believe less fluid is required during episode				
					Food: 44 % believe less food is required				
Rasheed et al., 1993 [117]	Saudi Arabia	Quantitative study: Structured interview (240) and self-administered questionnaire (589), Eastern Province 1990, Mothers of children attending government health center and girls attending government high school							Feeding: Fewer mothers than female students believe fluid and foods should be restricted during episode
									Drugs: Compared to students, more mothers preferred drugs as treatment
Africa									
Kaltenthaler et al., 1996 [30]	Botswana	Qualitative study: Focus group discussions FGDs (4) and IDIs (12), KIIs (7) and observations, North-east Botswana 1991–1992, Caregivers of young children, health providers and traditional healers				BF: Pogwana (severe diarrhea with sunken fontanel) is an “African illness” and should be treated with breast feeding cessation and should go to health facility or traditional healer			General: Mothers report using multiple sources of treatment if episode doesn’t improve
Nkwi et al., 1994 [34]	Cameroon	Mixed-method study: Structured interviews (256) and hospital observations, 3 provinces in Cameroon, Caregivers of children <5 years				BF: Some diarrhea thought to be caused by “bad breastmilk” - mothers are given herbs to improve quality of milk			
Almroth et al., 1997 [36]	Lesotho	Qualitative study: FGDs (19) and IDIs (43), 3 geographically different locations 1991–1992, Mothers and grandmothers of children and nurses	General: Mothers received conflicting advice from grandmothers and nurses	Food: Believe food should be given because it “strengthens the bowels”	Food: Believe you should adjust diet for individual child, if a specific food makes diarrhea worse				Food: Mothers coax children to eat during and after diarrhea
			Feeding: Caregivers report providers still advise caregivers to restrict feeding						General: Mothers report using any treatment that works, sometimes multiple treatments
Munthali et al., 2005 [35]	Malawi	Qualitative study: IDIs and KIIs (sample size not specified), Rumphi 2000–2002, Old and young men and women and health providers				BF: Perceived causes of diarrhea include contaminated breast milk requires weaning	Drugs perceived to useful in treatment of all illnesses		
						General: Diarrhea due to teething is perceived as requiring no treatment			
Ellis et al., 2007 [78]	Mali	Mixed methods study: Structured interviews (352), illness narratives (14), and IDIs (42), Bougouni District 2003, Caregivers of children <5 years with illness in past 2 weeks or seeking care and health providers	General: Mothers-in-law play important role initiating traditional treatment				Combining several different medicines/therapies is viewed as most efficacious	Treatment of diarrhea typically begins in the home with traditional medicines and/or antibiotics from nearby vendors	
Ikpatt et al., 1992 [19]	Nigeria	Quantitative study: Self-administered questionnaire (561), Cross River and Akwa Iborn State (year not specified), Household representative			BF: 19 % mothers believe BF should be discontinued				Drugs: 53 % of mothers reported antibiotic and 15 % reported antidiarrheal as treatment for diarrhea
					Fluid: 15 % believe fluid should not be offered during episode				
					Food: 17 % believe solid foods should be withdrawn				
Jinadu et al., 1996 [48]	Nigeria	Mixed method study: Structured interview (335) and FGD (4), Rural Yoruba communities of Osuo State (year not specified), Mothers of children <5 years				Fluid: More mothers believe fluids should not be given for watery diarrhea (65 %) compared to bloody diarrhea (55 %)			
Ogunbiyi et al., 2010 [29]	Nigeria	Mixed method study: Structured interviews (250) and FGDs (2), Ibadan 2003–2004, Mothers of child <1 year attending sick baby/immunization clinic of 2 health facilities	BF: “Cultural” reasons for BF restriction - passed from generations	Food: Foods withdrawn because thought to prolong the duration of diarrhea in the child (86 %) and induce vomiting/loss of appetite (14 %)	Food: Indigenous foods rich in protein withdrawn because believed to aggravate diarrhea	BF: Overconsumption of BM thought to cause some diarrhea – therefor reduce BF frequency during episode			
					Feeding: 71 % believe some food, fluid, or breast milk should be withdrawn during episode	Food: Withdrawal of other foods also linked to mother’s perception of cause of diarrhea			
Olakunle et al., 2012 [56]	Nigeria	Quantitative study: Structured interview (186), Ilorin West Local Government Area (year not specified), Mothers of children <5 years	Feeding: Majority said food restriction was based on personal view, but some said received information on food restriction from nurses		Feeding: 46 % of mothers believe “some food” should be restricted during episode				Drug: 17 % of mothers believe child should be treated with antibiotic during episode
Kauchali et al., 2004 [32]	South Africa	Qualitative study: IDIs (16), FGD (1), Case histories (13) and card sorting, Rural Kwazulu-Natal 2001, Caregivers of young children, grandmothers, CHWs				BF: Perceived causes of diarrhea include “dirty” breast milk requires temporary stop in breastfeeding			
Friend du Preeze et al., 2013 [72]	South Africa	Mixed method study: IDIs (17), FGDs (5) and structured interviews (206), Johannesburg and Soweto 2004, Caregivers of children <6 years in longitudinal study and health providers							Drugs: Health care workers reported that mothers commonly use non-prescribed antibiotics
									Drugs: Demand for modern medicines is high
Mwambete et al., 2010 [118]	Tanzania	Qualitative study: Semi-structured interviews (88), Dar es Salaam 2007, Mothers of children <5 years					35 % of mothers reported metronidazole as most effective chemotherapeutic agent for treating diarrhea		Drugs: Metronidazole (43 %) and Erythromycin + Metronidazole (12 %) were cited as commonly used “therapeutic agents” for diarrhea treatment
South East Asia									
Mushtaque et al., 1991 [55]	Bangladesh	Qualitative study: “Socioanthopologic methods,” Central Bangladesh (year not specified), villagers			Food: Certain types of diarrhea require withholding foods that are normally part of the diet	General: Treatments considered appropriate depend on the local classification of the diarrhea			
						BF: Injection of breast milk into woman used to correct “polluted” breast milk			
Singh et al., 1994 [43]	India	Quantitative study: Structured interviews (208), Jaipur District (year not specified), Mothers of children <5 years			Feeding: Mothers believe intestine becomes weak and child unable to digest heavy foods (roti and milk) during episode				
					Feeding: Tea water and banana believed to help reduce frequency of diarrhea				
Chandrashekar et al., 1995 [25]	India	Qualitative study: Semi-structured interviews (300), Rural South India 1991, Mothers of children age 3 days - 17 months	Feeding: Elderly relatives are source of information on feeding practices						BF: Some caregivers believe breastfeeding should be restricted when mother is experiencing diarrhea or respiratory infection
Buch et al., 1995 [119]	India	Quantitative study: Structured interview (1600), Kashmir 1992, Caregivers of infants with acute diarrhea attending hospital pediatric OPD			Feeding: 19 % of caregivers believe child should have complete dietary restriction				Drugs: 55 % of caregivers believe diarrhea should be treated with antidiarrheal & antispasmodic drugs, while 32 % should be treated with drugs and ORT
					Fluid: 77 % believe milk should be restricted				
Bhatia et al., 1999 [54]	India	Quantitative study: Structured interview (120), Rural Chandigarh 1996, Mothers of children <5 years			Feeding: 47 % of mothers believe certain foods/fluids should be restricted including chapatti, milk and pulses				
Datta et al., 2001 [120]	India	Quantitative study: Structured interview (75), Rural Maharashtra 2000, Caregivers of children <5 years attending hospital pediatric OPD			BF: 16 % of caregivers not aware child has to be given breastfeeding during episode of diarrhea				
Vyas et al., 2009 [121]	India	Quantitative study: Structured interview (380), Ganhinagar district (year not specified), Women of reproductive age (15–44)			BF: 52 % of women did not know breastfeeding should be continued during episode				
					Food: 50 % did not know other foods should be continued				
Bolam et al., 1998 [122]	Nepal	Quantitative study: Structured interview (105), Kathmandu 1994–1996, Women delivering at Kathmandu General Hospital			BF: 3 months postpartum, 53 % of mothers did not know to continue BF during episode				
Adhikari et al., 2006 [123]	Nepal	Quantitative study: Structured interview (510), Kathmandu 2005, Married women age 18–38 from 2 village development committees		BF: 7 % of women believe breastfeeding aggravates diarrhea					
Ansari et al., 2012 [24]	Nepal	Qualitative study: FGDs (2) and IDIs (8), Morang 2010, Mothers of children <45 months with diarrhea in the previous 6 months	General: Elders recommend traditional treatment practices		Food: Spicy, oily and rotten food commonly believed to be harmful	General: Certain types of diarrhea are perceived to be manageable with ORS/SSW, while others require traditional/spiritual methods.			
					BF: Breast milk sometimes considered harmful				
Baclig et al., 1990 [58]	Thailand	Mixed method study: FGDs (2) and structured interviews (98), Tambon Korat and Koongyang (year not specified), Mothers and grandmothers of children <5 years				Feeding: Mothers believe no changes should be made to the child’s diet to manage *poh* (a mild self-limiting diarrhea)			
Pylypa et al., 2009 [18]	Thailand	Qualitative study: Semi-structured interviews (200) as part of ethnographic study, Rural Northeast Thailand 2000–2001, Caregivers of children <5 years, traditional healers, and health providers	General: Grandmothers and elders are important sources of information for classifying/managing diarrhea	Fluid/BF: Some mothers restricted water or breast milk out of concern that it would make diarrhea worse, belief child could not drink much because he was small, or would vomit		Food: Most mothers didn’t change quantity/type of food given for diarrhea occurring in normal developmental stages (not illness) although expected children would eat less in than normal		Medicines were frequently obtained from health workers – most clinicians consulted gave antibiotics routinely for watery diarrhea, and for diarrhea with fever	Drugs: Some mothers took the medicines themselves to pass to infants through breast milk
						Drugs: Medicines were commonly administered for childhood diarrhea considered illness			
Western Pacific									
Okumura et al., 2002 [70]	Vietnam	Quantitative study: Structured interviews (505), 4 Provinces of Vietnam 1997, Mothers of children <5 years						Antibiotics to be stocked at home (55 % of households) for various anticipated symptoms as if they were panaceas	
Le et al., 2011 [69]	Vietnam	Qualitative study: IDIs (5) and FGDs (4), Ha Tay province (year not specified), Mothers of children <5 years and health workers/drug sellers	Drugs: Drugs bought on drug seller recommendation or previous prescriptions				Western medicine considered necessary but more dangerous than traditional therapy	Drugs are available without prescription and small amount can be purchased to give for 2–3 days	
Rheinlander et al., 2011 [67]	Vietnam	Qualitative study: Semi-structured interviews (43), FGDs (3), and observations, Ethnic minorities in Lao Cai 2008, Caregivers of children <7 years with diarrhea in the past month	General: Elders are in charge of deciding, preparing, and administering treatment for a sick child			Drugs: Medicines chosen based on perceived compatibility with the child and the disease	Antibiotics perceived as very powerful and potentially harmful compared to natural medicines		Drugs: common to receive 2–4 prescribed drugs for diarrhea
									Drugs: To limit intake and harm of western drugs, caregivers gave smaller doses than prescribed, or shifted from one drug to another if recovery was slow

Beliefs, motivations, and context related to:

BF: Breastfeeding

Fluid: Fluid restriction

Food: Food restriction

Feeding: Fluid, breastfeeding, and food restriction, or non-specific as to type of feeding

Drug: Use of modern medicines

General: Decision making around treatment or perception of diarrhea not specific to one of the harmful practice

### Breastfeeding reduction

Many studies reported the practice of breastfeeding reduction or cessation during diarrhea episodes (Table 1, Column 5). Most studies found that among mothers breastfeeding their child prior to the onset of diarrhea, fewer than 10 % of mothers stopped breastfeeding during the episode. The practice of breastfeeding cessation ranged from no mothers reporting breastfeeding cessation in a surveillance study in northeast Thailand to 62 % of mothers reporting stopping breast or milk feeding in a hospital-based study in Saudi Arabia [20, 28]. The practice of breastfeeding cessation was higher in hospital samples compared to samples from the general population. Where breastfeeding reduction was reported, on average one quarter of mothers reported reducing breastfeeding, although there was significant variation in the practice.

Multiple studies assessed variance in breastfeeding restriction by factors including characteristics of the diarrhea episode, child, caregiver, and household (Table 2). One study found younger and less educated mothers were more likely to reduce breastfeeding during episodes of diarrhea [12].

Mothers reported ceasing or reducing breastfeeding when their child had diarrhea for various reasons (Table 3). Mothers reported stopping or reducing breastfeeding because of beliefs that breastmilk was too fatty to be digested [20]. Others reported continued breastfeeding would not reduce the duration of diarrhea [20, 29] or could cause or worsen the diarrhea [18, 19, 29]. Caregivers in two studies believed specific types of diarrhea must be treated with breastfeeding cessation [29, 30]. In multiple cultures, “dirty” breast milk or secretion of ingested food through breast milk was thought to cause certain types of diarrhea. Mothers received treatment or a modified diet to improve the quality of their breast milk [31–34] or children were weaned [35]. Some caregivers stated they were following the advice of healthcare providers by restricting breastfeeding [20, 36]. Older relatives were also important sources of information on feeding practices during diarrhea episodes [25, 31]. In some studies, mothers continued feeding but diluted milk or formula [29], switched to powdered or goat’s milk [37], or only gave water [38].

### Food restriction

The measurement of food restriction, and prevalence estimates, varied widely across studies (Table 1, Column 6). Many studies differed in their definition or failed to specify if food restriction was measured only among those eating solid foods prior to illness, whether breastfeeding was included or excluded, and whether amount of food offered versus consumed was measured. Findings on restriction of specific foods have been included for context but not in prevalence estimates of overall food restriction (Table 1). The practice of stopping all food ranged from as low as 3 % of mothers stating they stopped giving solid or semi-solid foods during the episode in Oyo State, Nigeria [26] to as high as 53 % of mothers reporting they stopped feeding in Kenya [39]. As expected, measures that included the reduction of feeding in addition to complete restriction of feeding showed higher rates of food restriction, mostly within the range of 30–60 % of episodes.

Multiple studies addressed the variance of food restriction by other factors, including characteristics of the diarrhea episode, child, caregiver, and household (Table 2). Food curtailment was associated with dehydration and more severe disease [40], seeking care outside of the home, and ORS use [41]. In one study, caregivers were more likely to withhold food if a child had fever or a low appetite [11]. Another study found children less than 2 years of age were more likely to receive continued feeding compared to older children [42]. Two studies found that less educated mothers were more likely to restrict foods [12, 16].

Motivation for food restriction differed (Table 3). Some caregivers reported that a child’s diet should be restricted because of beliefs that a child cannot eat or digest as much during a diarrhea episode [22, 43] and feeding can exacerbate or prolong diarrhea episodes [19, 22, 29, 44–46]. Belief that only certain foods should be restricted because they can aggravate diarrhea was common across countries and included a range of foods such as meat, milk, sweet food, greasy food, high carbohydrate and high protein foods [29, 37, 38, 43, 47–54]. Alternatively, in two studies some caregivers reported that specific foods were customary and should be given during a diarrhea episode to strengthen the bowel or soothe the stomach [36, 52]. Some caregivers reported that restriction of certain foods was based on long held folk tradition [29, 47]. Others reported that diet alteration is based on the type or perceived cause of the diarrhea [18, 29, 55]. Elderly relatives, neighbors, and health care providers were reported to influence mothers’ feeding practices in many contexts [22, 23, 25, 27, 29, 36, 53, 56, 57]. Some caregivers reported that a child’s diet was not restricted during diarrhea because it was already limited [27, 44, 58]. One study reported mothers coaxed their child to eat more [36], but others reported some mothers of children with decreased appetite were unfamiliar with encouraging children to eat [22, 44] or had little time to prepare additional food because they were caring for the child [22]. One study suggested caregivers felt continued feeding was less important if they had been given some treatment at a health facility [31].

### Inappropriate medication use

Many studies reported the use of drugs to treat diarrhea in children under five (Table 1, Column 7). The most commonly reported measures were the use of an antibiotic or antimicrobial, followed by use of any medicine, and the use of an antidiarrheal or antimotility agent. While antibiotics are recommended for treatment of dysentery or cholera, most studies did not differentiate between simple and dysenteric diarrhea when reporting on antibiotic use. The Lives Saved Tool (LiST) attributes 7 % of diarrhea cases in children under 5 to dysentery [59], therefor it may be inferred that high antibiotic use rates are inclusive of inappropriate antibiotic use. A hospital-based study in Enugu, Nigeria highlights the difficultly of collecting information on the type of medicine used to treat diarrhea. The study reported that 70 % of mothers misclassified antibiotics and analgesics as antimotility agents when self-reporting drugs used in diarrhea treatment [60]. Multiple studies outside of this review have shown that the accuracy of drug recall varies by questionnaire design and method of assessment [61].

Reported use of antidiarrheal and antimotility agents was generally lower than reported use of antibiotics. Use of antibiotics at any point in an episode ranged from 10-77 %. Antidiarrheal use ranged from 3–45 % of diarrhea episodes, with the exception of very high reported use (74 %) in Egypt in 2002 [62]. Use of any drug for a diarrhea episode occurring in the previous 2 weeks ranged from 26–76 %. Studies that used a shorter reference period limited to the previous 24 h reported lower rates of drug use at around 20 %.

Multiple studies addressed variance in inappropriate medication use by factors including characteristics of the diarrhea episode, child, caregiver, and household (Table 2). A hospital-based study in Nigeria found children who had received an antibacterial or antidiarrheal at home presented to the hospital with more severe dehydration than those children who did not receive these drugs [60]. Antibiotic and/or antidiarrheal use were associated with seeking care outside of the home [11, 41] and use of ORT [60, 63]. Two studies in Enugu, Nigeria reported conflicting associations between maternal education and antibiotic use [60, 64].

Caregivers reported using antibiotics and other drugs to treat diarrhea because they were accessible and believed to be efficacious (Table 3). Multiple studies reported caregiver beliefs that modern medicines are powerful [64–67], and more effective in treating diarrhea than ORS [65, 68]. Multiple studies reported drugs were widely available and affordable in the public and private sector, typically without prescription [35, 38, 40, 44, 49, 52, 64, 69]. In many contexts, caregivers stocked drugs at home, purchasing them in advance or saving leftover medication from previous illnesses [33, 37, 38, 52, 70]. Caregivers perceived drugs to be cheaper and more accessible than ORS, particularly given the flexibility to purchase a few tablets for little money [64, 65, 71]. Use of antibiotics in the treatment of pediatric diarrhea has become routine for both health care providers and caregivers in some contexts [18, 40, 66]. Caregivers may have also influenced provider behavior as caregivers’ preference for drug therapies creates pressure on providers to give medications in addition or instead of ORS [28, 33, 65, 72]. Drugs were given in sub-clinical doses in multiple studies [67, 69, 73]. It was common in studies for children to receive multiple drugs for a single episode of diarrhea, often from the same source [67, 74–77]. A study in Brazil found drugs were used more commonly to treat episodes of longer duration [63], although initial treatment of diarrhea at home with drugs was common in a study in Mali [78]. Multiple studies suggested treatment with modern medicines may be related to the perceived cause or type of diarrhea [18, 52, 60, 79–81]. Treatment seeking was often related to inappropriate use of medicine for diarrhea management [33, 57, 62, 82].

## Discussion

This is the first review, to our knowledge, that addresses harmful practices related to fluids, feeding and medication use during episodes of childhood diarrhea. The findings indicate that there have been many studies – both quantitative and qualitative – that have documented these harmful practices. However, reported prevalence varies greatly across study populations, and we were unable to identify clearly defined patterns across regions, countries, or time periods. A limited number of studies looked at the variation of these harmful practices across potential influencing factors, including characteristics of the diarrhea episode and child, caregiver, or household-level traits. Findings of association differed across studies.

The motivation for harmful practices during diarrhea treatment also appears to vary across populations, although studies consistently report general caregiver concern for their child’s health and caregiver action to treat the illness to the best of their knowledge and abilities. Caregivers reported that their actions were based on the advice of health care providers, community members, or elderly relatives, as well as their own observations or understanding of the efficacy of certain treatments for diarrhea. Others reported following traditionally held beliefs on the causes and cures for specific diarrheal diseases.

Across studies, the measurement of harmful practices was inconsistent and not guided by a conceptual or theoretical framework. Most studies were focused on general practices in diarrhea treatment, and harmful practices were rarely a primary outcome of interest. This has limited the availability and quality of data on the topic. Variations in study design, sample populations, diarrhea episode reference periods, and measurement definitions make drawing comparisons and conclusions across studies challenging. This is further compounded by inconsistent quality in data collection and reporting. Most studies relied on sub-national population samples and many were limited to small sample sizes. The variation in treatment practices by perceived type of diarrhea highlights the importance of using local terminology in order to capture all episodes of diarrhea as perceived by the community [83]. Although the majority of studies included in this review used a recall period of diarrhea in the past two weeks, there was some variation ranging from the past 24 h to past six months or the “most recent” episode of diarrhea. Fischer-Walker and her colleagues highlight the importance of using a shorter recall period for capturing episodes of diarrhea of varying severity [83].

Although this systematic review highlighted limitations of existing research, the available evidence suggests that harmful practices in diarrhea treatment are common in certain populations. A multicountry analysis using MICS data from 28 countries between 2005–2007 reported the majority of mothers did not maintain their child’s nutritional intake during illness [5]. Analysis of DHS data from 14 countries between 1986–2003 suggests a decreasing trend in continued feeding in a majority of countries [6]. These practices can reduce correct management of diarrheal disease in children and result in treatment failure and sustained nutritional deficits. The lack of consistency in sampling, measurement, and reporting identified in this literature review highlights the need to document harmful practices using standard methods of measurement and reporting. Going forward, studies in this area would benefit from the development and use of a broader conceptual framework to ensure that the research is theory-driven and regularly synthesized. Multi-country analyses using MICS and DHS data have been conducted in the past, but they have tended to focus on positive treatment practices rather than harmful practices [5, 6]. Assessing harmful practices with nationally representative data and standardized measurements, through the analysis of the most recently available DHS and MICS data, can contribute to the discussion on improved care of diarrheal disease in children under five.

The strengths of this literature review include applying a systematic process for searching and summarizing the literature, and accessing articles during a time frame in which global efforts focused on improving coverage. This review was limited by the inclusion of only peer-reviewed literature and the exclusion of non-English language publications. Additionally, the quality of individual articles was not assessed, allowing for the potential inclusion of studies with misrepresentative findings.

## Conclusions

Harmful practices in the management of childhood diarrhea are prevalent to varying degrees across cultures and include fluid and breastfeeding curtailment, food restriction, and inappropriate medication use. Inappropriate management of diarrhea episodes can result in higher risk of mortality through increased levels of dehydration or lasting health consequences as a result of nutritional restrictions or prolonged diarrhea illness. These practices must therefore be addressed as a matter of urgency in maternal, newborn and child health programs. These programs need to target not only the behaviors of child caregivers, but the broader social network, because our findings show that these practices are often informed by traditional beliefs, popular knowledge, and the instruction of authority figures, including elderly community members and health workers. Broader health systems interventions are also needed to address the alarming findings of high rates of inappropriate use of medications during diarrhea episodes. In addition, the global health community must do a better job or measuring the prevalence of these practices in standard ways, to produce evidence that can be used as the basis for action.

## Additional file

Additional file 1:
**PubMed Search Terms.** (PDF 68 kb)
